# Supplementary material for: Managing energy infrastructure to decarbonize industrial parks in China
Source: Nat Commun. 2020 Feb 20;11:981. doi: 10.1038/s41467-020-14805-z (PMC7033157; doi:10.1038/s41467-020-14805-z)
Supplement: Supplementary file 1 — Supplementary Information [file 41467_2020_14805_MOESM1_ESM.pdf]

SUPPLEMENTARY INFORMATION

Managing Energy Infrastructure to Decarbonize Industrial Parks in China

Guo et al.

This file includes

Supplementary Figures 1-7,

Supplementary Tables 1-21,

Supplementary Notes 1-7,

and Supplementary References.

Supplementary Figures

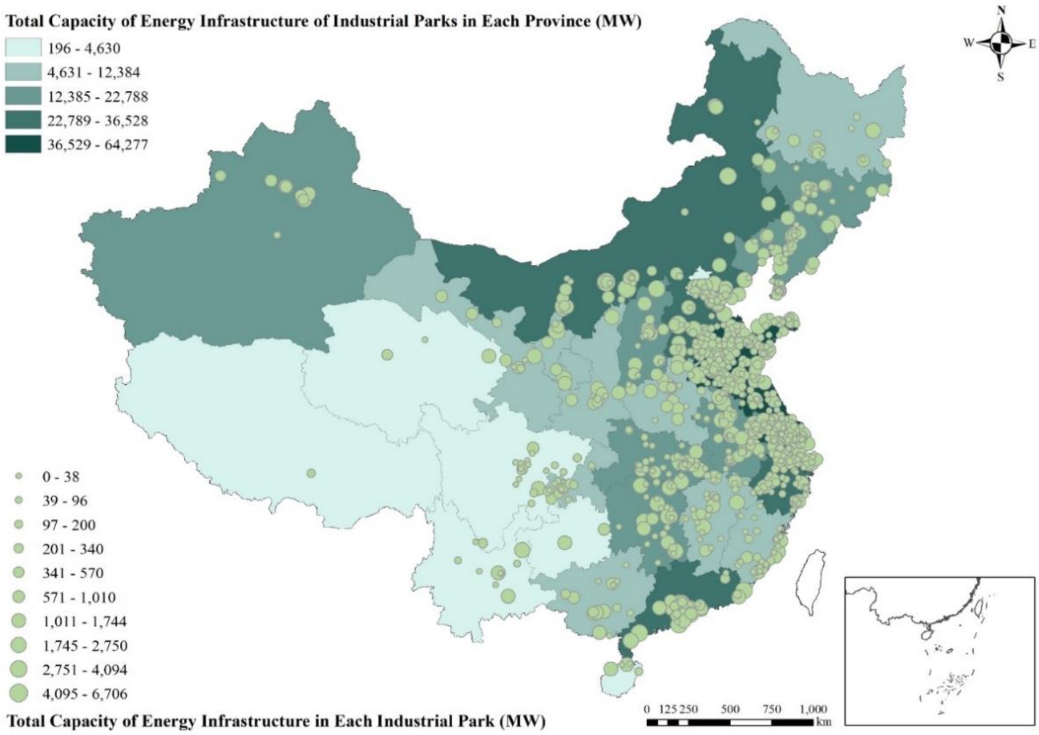

Supplementary Figure 1. Energy infrastructure capacity of the 1,604 Chinese industrial parks. Source data are provided as a Source Data file.

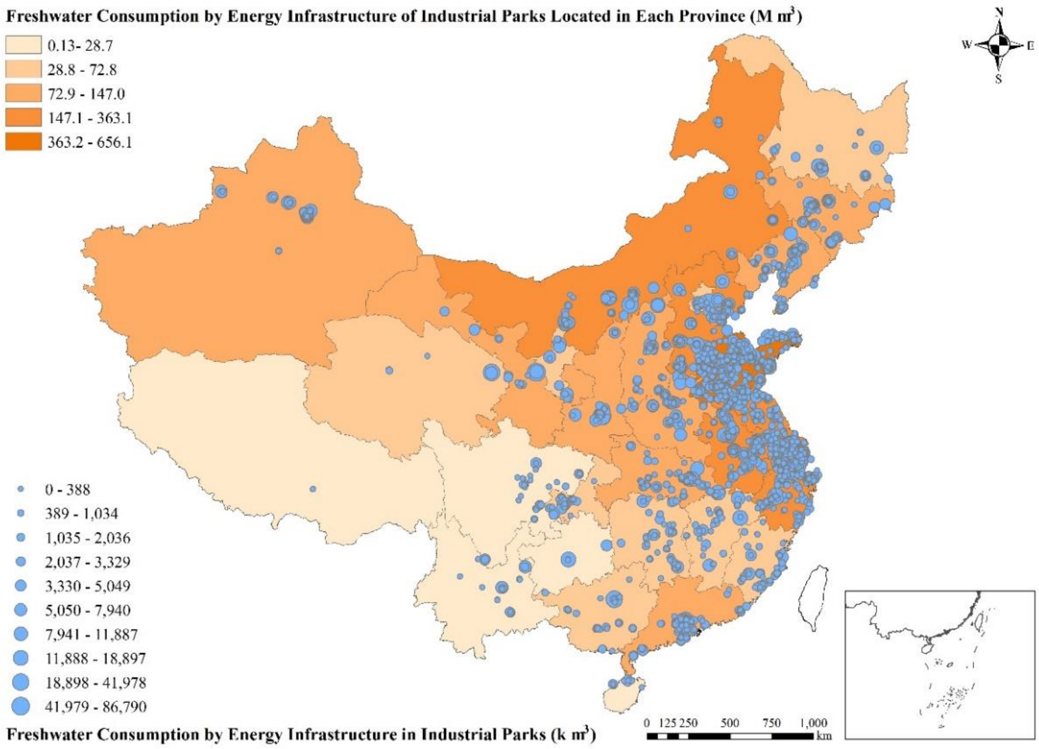

Supplementary Figure 2. Freshwater consumption by energy infrastructure in the 1,604 Chinese industrial parks. Source data are provided as a Source Data file.

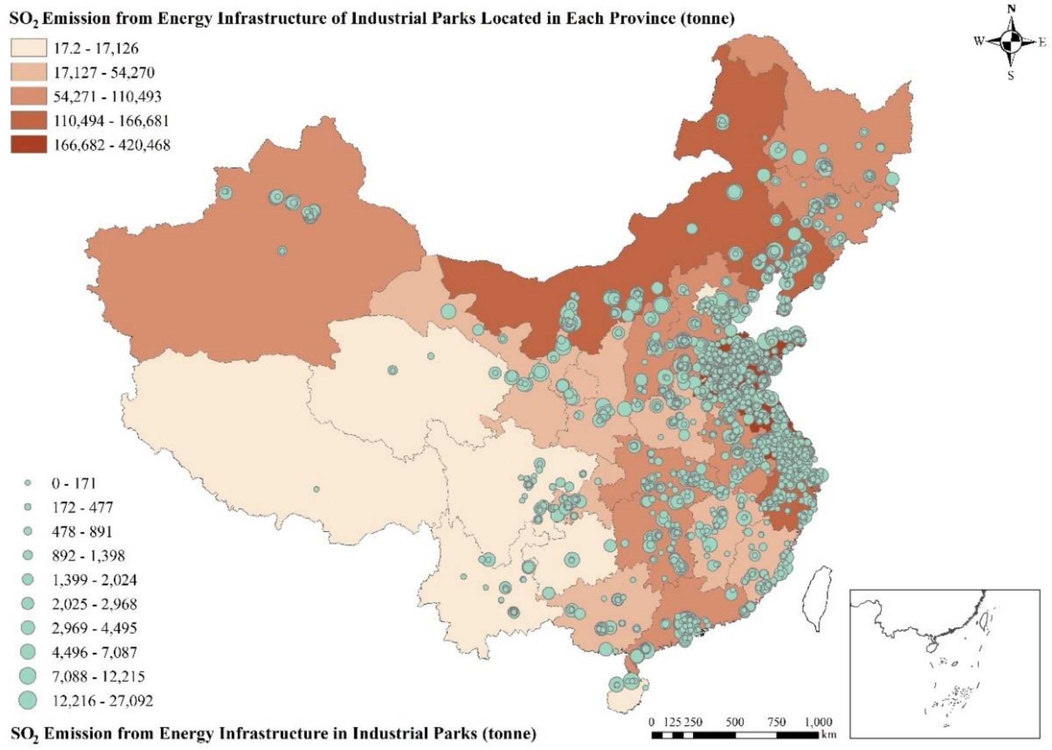

Supplementary Figure 3. SO_2 emission from energy infrastructure in the 1,604 Chinese industrial parks. Source data are provided as a Source Data file.

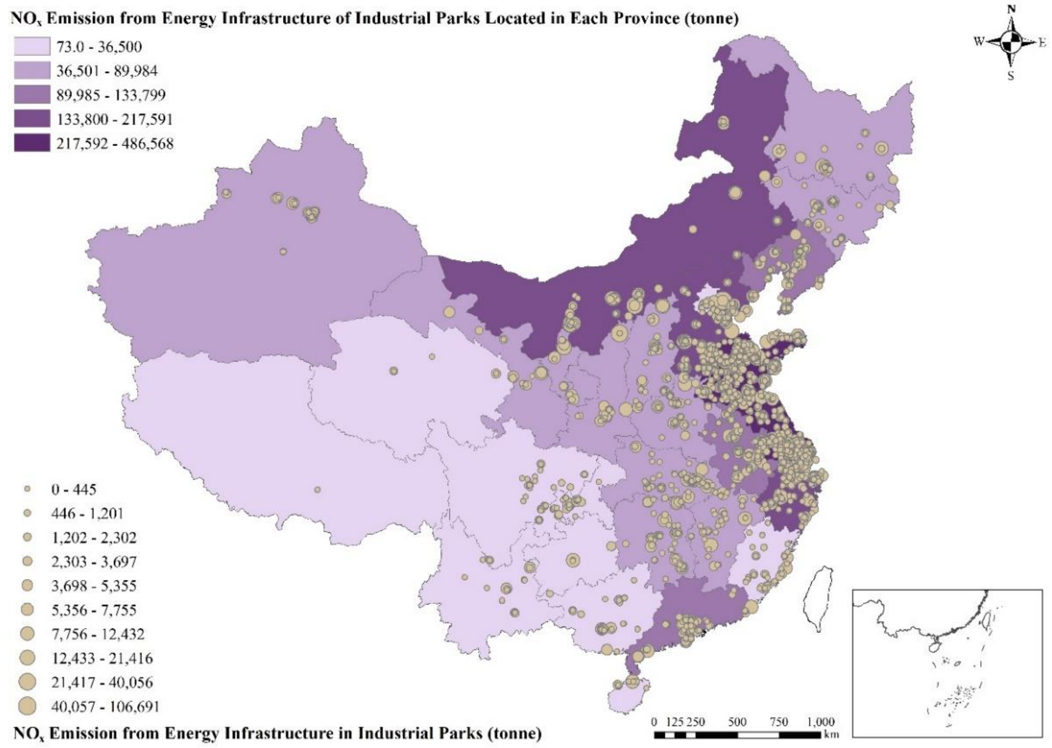

Supplementary Figure 4. NO_x emission from energy infrastructure in the 1,604 Chinese industrial parks. Source data are provided as a Source Data file.

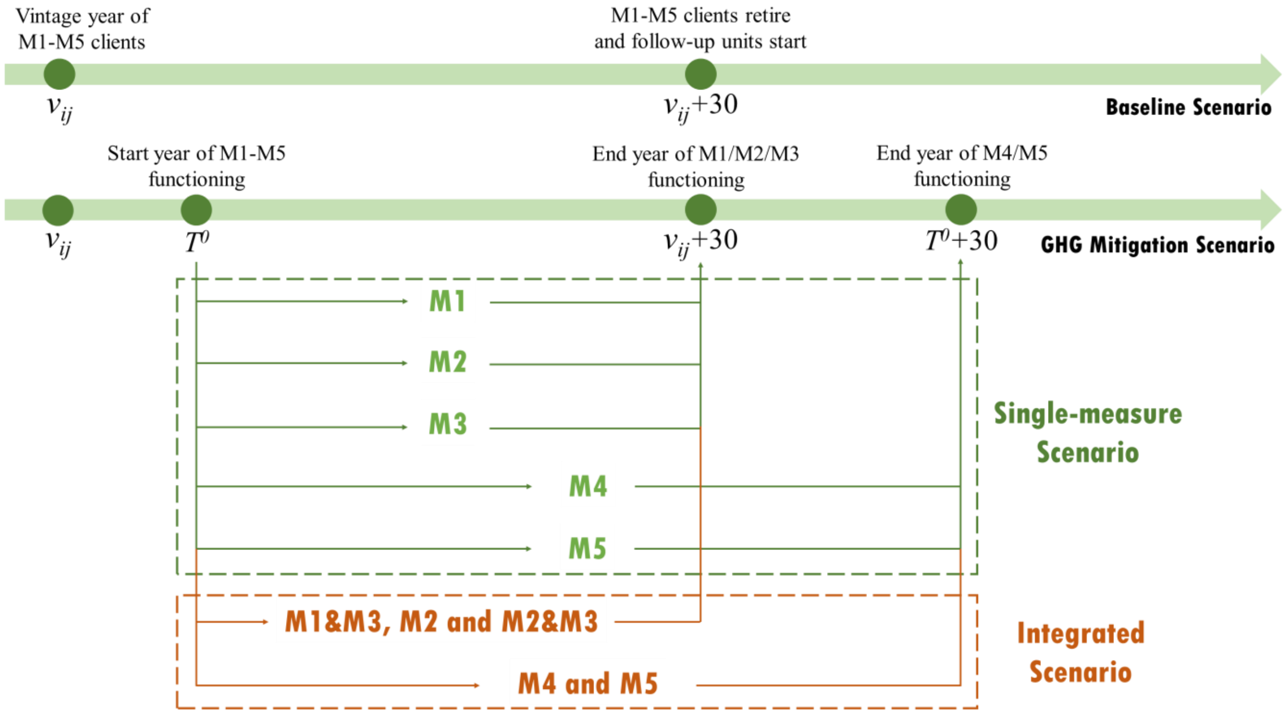

Supplementary Figure 5. Timeline of the baseline, M1-M5, and integrated scenarios.

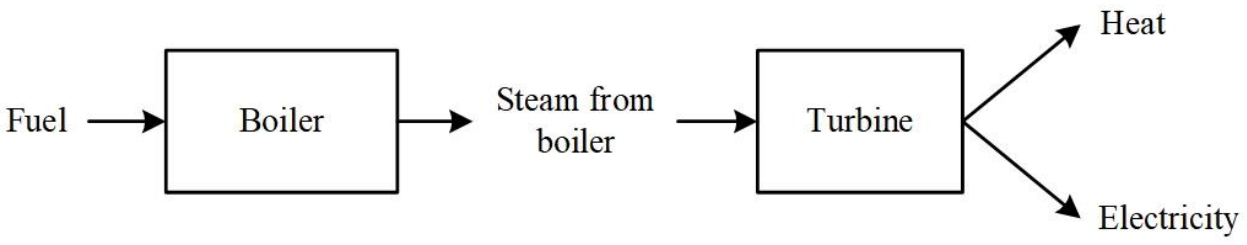

Supplementary Figure 6. Flow chart of the conversion from fuel input to energy outputs.

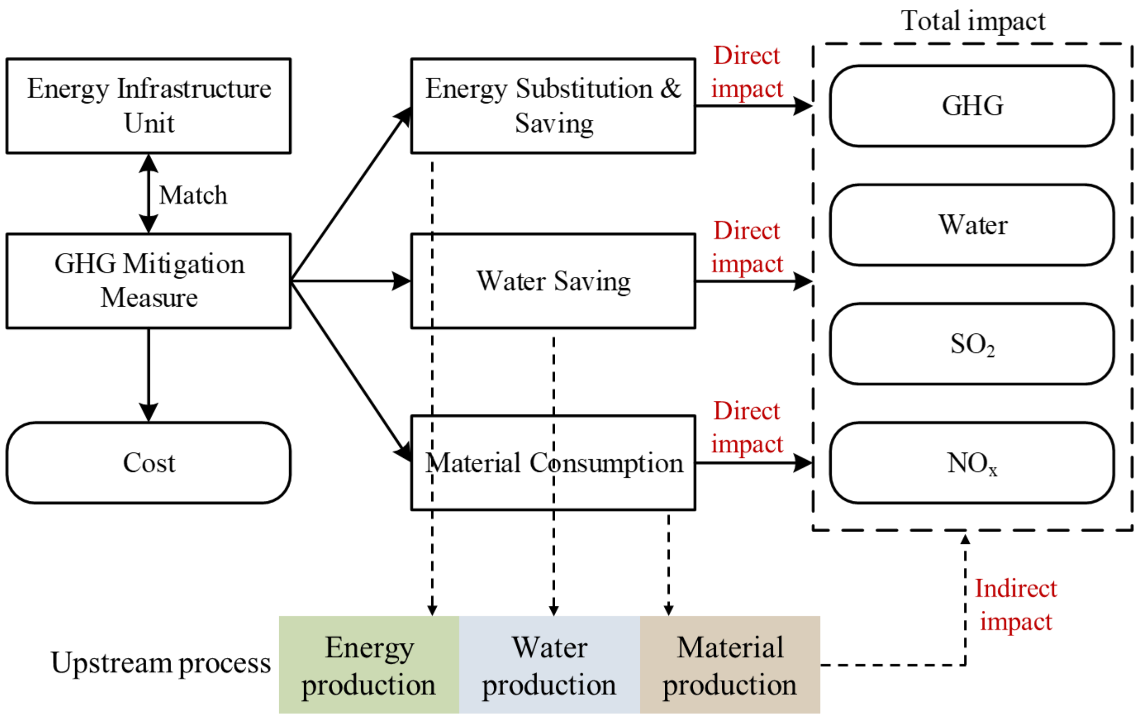

Supplementary Figure 7. Energy-water-material nexus based on a life-cycle thinking.

Supplementary Tables

Supplementary Table 1. Boiler efficiencies of energy infrastructure categorized by fuel

Fuel	Default	Minimum	Maximum	Reference
Coal / coal gangue	0.85	0.8	0.9	General Administration of Quality Supervision, Inspection and Quarantine of China, 2010. Supervision Administration Regulation on Energy Conservation Technology for Boiler (TSGG0002-2010).
Natural gas / coal gas	0.92	0.9	0.94	
Diesel	0.91	0.88	0.94	
MSW / biomass	0.725	0.6	0.85	Astrup, T., Møller, J., Fruergaard, T. Incineration and Co-combustion of Waste: Accounting of Greenhouse Gases and Global Warming Contributions. *Waste Management & Research*. 2009. 27(8), 789-799.
Ratio of mixed coal to total fuel input (in calorific value)	0.1	0	0.2	Ministry of Environmental Protection, 2006. Strengthening the Management of Environmental Impact Assessment of Biomass Power Generation Project. http://law.npc.gov.cn/FLFG/flfgByID.action?txtid=4&flfgID=230988&showDetailType=QW (Accessed Jan 1, 2019).
Waste heat	0.8	0.75	0.85	Walker, M., Lv, Z., Masanet, E. Industrial Steam Systems and the Energy-Water Nexus. *Environmental Science & Technology*. 2013. 47(22), 13060-13067.

Supplementary Table 2. Heat-to-electric ratios of energy infrastructure categorized by capacity

Unit capacity	Default (median)	Minimum	Maximum
≥600MW	0.022	0.0003	0.744
300≤ <600	0.229	0.0017	1.669
100≤ <300MW	0.469	0.0532	3.480
<100MW	4.321	0.4838	14.964

Note: The heat-to-electric ratios in this table are statistical values based on our database.

Supplementary Table 3. Effective electrical efficiencies, annual working hours, and self-use electricity rates of energy infrastructure categorized by fuel, capacity, and technology

Fuel	Capacity and technology	Effective electrical efficiency[a]			Annual working hours (h/a)			Self-use electricity rate (%)		
		Def[b]	Min[b]	Max[b]	Def	Min	Max	Def	Min	Max
Coal	PC[c], <100	0.286	0.130	0.433	3731	117	8859	10.5	0.1	65.4
	PC, 100≤ <300	0.352	0.296	0.431	4315	535	7226	8.7	3.9	12.8
	PC, 300≤ <600	0.378	0.334	0.442	4754	2045	8352	6.1	1.8	11.7
	PC, ≥600	0.399	0.346	0.460	4795	1237	7323	5.1	1.5	11.2
	EC[c], <100	0.368	0.121	0.793	4118	140	9875	8.8	0.1	46.4
	EC, 100≤ <300	0.380	0.271	0.585	5057	1223	9111	8.0	1.3	21.7
	EC, 300≤ <600	0.388	0.253	0.532				6.1	1.4	16.1
	EC, ≥600	0.403	0.375	0.434	5149	3556	7128	4.9	2.7	7.6
	BP[c]	0.494	0.228	0.884	4414	355	8760	7.6	1.6	33.4
Natural gas	NGCC[c]	0.481	0.302	0.690	3049	232	7633	3.4	0.8	35.5
	PC/EC	0.436	0.352	0.654				4.4	0.5	7.7
Coal gas	CGCC[c]	0.371	0.308	0.436	5491	2347	7329	8.2	6.2	10.5
	PC	0.386	0.162	0.621	4737	416	9029	11.5	1.1	35.5
	EC	0.318	0.202	0.414				12.6	7.7	19.7
Coal gangue	PC	0.307	0.218	0.398	4185	189	8765	9.6	5.1	14.7
	EC	0.313	0.186	0.385	4977	584	7786	11.3	0.8	22.3
Diesel	PC	0.275	0.104	0.377	2433	727	6044	14.9	3.4	33.2
	EC	0.355	0.228	0.459	3058	1441	4661	7.5	1.6	13.2
MSW	PC	0.231	0.137	0.378	5518	858	8766	16.6	5	34.4
	EC	0.344	0.156	0.679	5658	610	9223	13.9	4.4	29.3
Biomass	PC	0.272	0.216	0.354	5992	77	8478	8.1	0.7	19.5
	EC	0.290	0.201	0.572	5702	272	9125	10.6	4	31.6
	BP	0.541	0.369	0.698	3645	1197	5033	11.1	4.9	21.7
Sludage	EC	0.335	0.271	0.383	6422	4846	9070	6.0	2.7	7.9
	BP	0.467	0.414	0.521	2753	2275	3230	5.5	3.5	7.4
Waste heat	PC	0.355	0.207	0.621	4970	416	9029	8.2	0.6	76
	EC	0.342	0.235	0.516	4118	565	8647	11.6	4	23
	BP	0.595	0.396	0.793	5147	2056	9029	4.3	3.6	4.9

Note:

a) Effective electrical efficiency (EEE) is a more useful than overall energy efficiency for fuel saving evaluation. According to combined heat and power (CHP) guidelines[3], *EEE* is calculated assuming the heat output from CHP systems would otherwise be generated by industrial boilers driven by the same fuel as CHP plants.

b) Def = default (taking average value), Min = minimum, and Max = maximum.

c) PC = pure condensing, EC = extraction condensing, BP = back pressure, NGCC = natural gas combined cycle, and CGCC = coal gas combined cycle.

Supplementary Table 4. Uncertainty analysis of reduction rates in GHG emission, freshwater consumption, SO2 emission, and NOx emission

Valuation	Scenario	GHG	Water	SO_2	NO_x
Original results for GHG mitigation rates	M1	-0.68%	0.27%	-2.52%	-1.64%
	M2	-2.71%	2.49%	-6.55%	0.84%
	M3	-4.99%	-30.94%	-8.45%	-5.92%
	M4	-2.60%	-12.95%	-18.44%	-8.17%
	M5	-2.61%	-3.84%	-6.72%	-3.24%
	Integrated	-10.15%	-37.93%	-26.09%	-10.63%
Minimum results for GHG mitigation rates	M1	-0.44%	0.26%	-2.00%	-1.13%
	M2	-1.15%	2.77%	-3.22%	0.62%
	M3	-4.44%	-31.82%	-8.68%	-5.36%
	M4	-1.38%	-13.71%	-14.38%	-4.27%
	M5	-2.63%	-4.08%	-8.02%	-3.32%
	Integrated	-8.04%	-39.20%	-23.76%	-9.68%
Maximum results for GHG mitigation rates	M1	-1.03%	0.25%	-2.93%	-2.26%
	M2	-5.62%	1.45%	-11.00%	0.67%
	M3	-7.06%	-27.28%	-9.73%	-8.26%
	M4	-5.77%	-11.11%	-25.48%	-15.18%
	M5	-2.42%	-3.22%	-4.61%	-2.90%
	Integrated	-15.93%	-33.65%	-30.59%	-13.77%

Supplementary Table 5. Key variables and parameters in the vintage stock model

Variable/parameter	Definition
Num_i	Number of units belonging to the ith plant, *i=1…2,127*
Cap_{ij}	Capacity of the jth unit of the ith plant (MW), $j=1\ldots Num_i$
EO_i	Annual electricity output of the ith plant (GJ)
HO_i	Annual heat output of the ith plant (GJ)
SFB_i	Steam from boiler of the ith plant (GJ)
FFE_i	Fuel for electricity of the ith plant (GJ) based on a low calorific value (see Supplementary Table 6)
FFH_i	Fuel for heat of the ith plant (GJ) based on a low calorific value (see Supplementary Table 6)
EE_i	Energy efficiency of the ith plant
EEE_i	Effective electrical efficiency of the ith plant
BE_i	Boiler efficiency of the ith plant
$(H/E)_i$	Heat-to-electric ratio of the ith plant
$WorkTime_i$	Annual working time of the ith plant (hours)
$SelfUseRate_i$	Self-use electricity rate of the ith plant
$RemLife_{ij}$	Remaining serviceable lifetime of the jth unit of the ith plant (a)
$Fuel_{ij}$	Fuel category of the jth unit of the ith plant
$TurTech_{ij}$	Turbine technology of the jth unit of the ith plant
$CoolTech_{ij}$	Cooling technology of the jth unit of the ith plant
$Prov_i$	Province the ith plant is located in
$GHGFac$	GHG emission factor (tCO_2 eq./GJ, see Supplementary Table 7)
$WatFacEle$	Freshwater consumption factor for electricity generation (m^3/GJ, see Supplementary Table 8)
$WatFacHeat$	Freshwater consumption factor for heat generation (m^3/GJ, see Supplementary Table 9)
$SO2Fac$	SO_2 emission factor (t/GJ, see Supplementary Table 10)
$NOxFac$	NO_x emission factor (t/GJ, see Supplementary Table 10)
$MixRate$	Mixed rate of coal to total fuel input into incinerators based on a low calorific value
$GHGEmi$	GHG emissions of all units (t CO_2 eq.)
$WaterCon$	Freshwater consumption of all the units (m^3)
$SO2Emi$	SO_2 emissions of all units (t)
$NOxEmi$	NO_x emissions of all units (t)
$GHGMit$	GHG emission mitigation of all units (t CO_2 eq.)
$WaterSav$	Freshwater consumption saving of all the units (m^3)
$SO2Red$	SO_2 emission reductions of all units (t)
$NOxRed$	NO_x emission reductions of all units (t)
BA/IN	Baseline or integrated scenario (superscript of relevant variables)
D/I	Direct or indirect environmental impact (subscript of relevant variables)

Supplementary Table 6. Lower calorific value of each energy category

Category	Unit	Lower calorific value based on standard coal equivalent
Coal	kgce/kg	0.7143
Diesel		1.4571
Coal gangue		0.2857
Municipal solid waste/sludge		0.2714
Biomass		0.5
Natural gas	$kgce/m^3$	1.33
Coke oven gas		0.5714
Blast furnace gas		0.1286
Biogas		0.714

Note: kgce is short for *kg standard coal equivalent*. 1 kgce=29.27 MJ. The data on lower calorific value is sourced from National Guideline for Energy Consumption Accounting of China (GB/T2589-2008), and 2017 Guideline for Energy Consumption Accounting of Shanghai City (http://www.stats-sh.gov.cn/html/tjfw/201801/1001456.html).

Supplementary Table 7. GHG emission factors of each energy category

Fuel	tCO_2/GJ	gCH_4/GJ	$g\ N_2O/GJ$	tCO_2 eq./GJ
Coal	0.0948	1.0000	1.5000	0.0952
NG	0.0555	1.0000	0.1000	0.0556
Diesel	0.0726	3.0002	0.6000	0.0728
MSW/sludge	0.0330	-	-	0.0330
Coal gangue	0.0946	-	-	0.0946
Biomass/biogas	-	30.0376	4.0048	0.0019
Coke oven gas	0.0493	1.0001	0.1000	0.0494
Blast furnace gas	0.2599	1.0012	0.1001	0.2600

Note： All the values in this table refers to mineral emission, excluding biogenic emission. CO_2, CH_4, and N_2O emission factors are cited from GHG Protocol Tool for Energy Consumption in China (V2.1) issued by World Resources Institute. CO_2, CH_4, and N_2O are converted into CO_2 equivalent according to 100-year global warming potential with the ratios of 1, 28, and 265, issued by IPCC Working Group I in *Climate Change 2013: The Physical Science Basis*.

Supplementary Table 8. Freshwater consumption factors of electricity generation

Category	Freshwater consumption factor (m^3/MWh)		
	Once through	Recirculating	Dry
Coal-fired, 1000 MW	0.228[a]	1.688[b]	0.31[a]
Coal-fired, 600 MW	0.28[a]	1.65[b]	0.334[a]
Coal-fired, 300 MW	0.343[a]	1.89[b]	0.417[a]
Coal-fired, 100~225 MW	0.556[a]	2.16[b]	0.59[a]
Coal-fired, <100 MW	0.556[b]	2.47[b]	0.59[a]
Natural gas-fired, steam cycle	-	2.76[c]	-
Natural gas-fired, combined cycle	0.379[c]	0.795[c]	-
Coal gas-fired, steam cycle	-	3.22[b]	-
Coal gas-fired, combined cycle	-	1.68[b]	-
Biomass	-	3.63[b]	-
Municipal solid waste	-	6.36[b]	-
Waste heat	-	9.61[b]	-

Note: Freshwater consumption factors of electricity generation include that for cooling and other processes, e.g., boiler water makeup, slag removal, and flue gas desulfurization. The factors of part units were collected from basic information tables for the thermal power-generating units issued by China Electricity Council (Refs 53-55 in the main text), project reports, and official websites of power plants. The factors of the other units were supplemented by the default values in this table.

Sources:

[a]China Electricity Council. Basic Information Tables for Thermal Power Generating Units (2011). *China Electricity Council: Beijing*. 2012.

[b]Zhang, C., Zhong, L., Fu, X., Wang, J., Wu, Z. Revealing water stress by the thermal power industry in China based on a high spatial resolution water withdrawal and consumption inventory. *Environmental Science & Technology*. 2016, 50(4), 1642-1652.

[c]Meldrum, J., Nettles-Anderson, S., Heath, G., Macknick, J. Life cycle water use for electricity generation: a review and harmonization of literature estimates. *Environmental Research Letters*. 2013, 8, 015031.

Supplementary Table 9. Freshwater consumption factors of heat generation

Provincial-level administrative region	Freshwater consumption factor (m^3/GJ)	Provincial-level administrative region	Freshwater consumption factor (m^3/GJ)
Beijing	0.0178	Henan	0.0825
Tianjin	0.0646	Hubei	0.0532
Hebei	0.0869	Hunan	0.0390
Shanxi	0.1156	Guangdong	0.0336
Inner Mongolia	0.1496	Guangxi	0.0287
Liaoning	0.1167	Hainan	0.0253
Jilin	0.1112	Chongqing	0.0490
Heilongjiang	0.0589	Sichuan	0.0404
Shanghai	0.0246	Guizhou	0.0517
Jiangsu	0.0756	Yunnan	0.0603
Zhejiang	0.0490	Shaanxi	0.0896
Anhui	0.0850	Gansu	0.1079
Fujian	0.0471	Qinghai	0.0728
Jiangxi	0.0542	Ningxia	0.1239
Shandong	0.1022	Xinjiang	0.1089

Source: Zhang, C., Anadon, L. Life Cycle Water Use of Energy Production and Its Environmental Impacts in China. *Environmental Science & Technology*. 2013, 47(24), 14459-14467.

Supplementary Table 10. SO_2 emission and NO_x emission factors of energy infrastructure units

Unit category	SO_2 emission factor		NO_x emission factor	
	Technology	kg/t	Technology	kg/t
Coal-fired, ≥750MW	Limestone-plaster	1.809	Low-nitrogen combustion, flue gas denitrification	2.13
Coal-fired, 450~749MW	Limestone-plaster	1.790	Low-nitrogen combustion, flue gas denitrification	2.12
	Seawater desulfurization	2.028	Low-nitrogen combustion, SNCR[b], emitting directly	4.25
Coal-fired, 250~449MW	Limestone-plaster	1.443	Low-nitrogen combustion, flue gas denitrification	2.04
	Seawater desulfurization	1.572	Low-nitrogen combustion, SNCR, emitting directly	4.07
	Flue gas CFB[a] desulfurization	1.572		
	CFB boiler, emitting directly	2.361		
Coal-fired, 150~249MW	Limestone-plaster	1.290	Low-nitrogen combustion, flue gas denitrification	1.96
	Seawater desulfurization	1.384	Low-nitrogen combustion, SNCR, emitting directly	3.93
	Flue gas CFB desulfurization	3.460		
	CFB boiler, emitting directly	4.153		

Unit category	SO_2 emission factor		NO_x emission factor	
	Technology	kg/t	Technology	kg/t
Coal-fired, 75~149MW	Limestone-plaster	1.288	Low-nitrogen combustion, flue gas denitrification	1.92
	Integration of wet dust removal and desulfurization	4.145	Low-nitrogen combustion, SNCR, emitting directly	3.84
	Seawater desulfurization	1.382		
	Flue gas CFB desulfurization	3.452		
	CFB boiler, emitting directly	4.145		
Coal-fired, 35~74MW	Limestone-plaster	1.273	Ammonia desulphurization, emitting directly	5.85
	Ammonia desulphurization	0.685	Ammonia desulphurization, low-nitrogen combustion	3.51
	Integration of wet dust removal and desulfurization	4.104		
	Flue gas CFB desulfurization	4.104		
	Spray drying / simple wet limestone-plaster	4.104		
	CFB boiler, emitting directly	4.790		
Coal-fired, 20~34MW	Integration of wet dust removal and desulfurization	4.142	Ammonia desulphurization, emitting directly	5.55
	Flue gas CFB desulfurization	4.142	Ammonia desulphurization, low-nitrogen combustion	3.33
	Ammonia desulphurization	0.689		
	Spray drying / simple wet limestone-plaster	4.143		
	CFB boiler, emitting directly	4.840		
Coal-fired, 9~19MW	Pulverized coal furnace, integration of wet dust removal and desulfurization	4.039	Pulverized coal furnace, ammonia desulphurization, emitting directly	4.37
	Pulverized coal furnace, ammonia desulphurization	0.677	Pulverized coal furnace, ammonia desulphurization, low-nitrogen combustion	2.62
	CFB boiler, emitting directly	4.708	Grate furnace, emitting directly	4.38
	Grate furnace, integration of wet dust removal and desulfurization	2.742	CFB boiler, emitting directly	3.63
Coal-fired, ≤8MW	Grate furnace, integration of wet dust removal and desulfurization	2.742	Grate furnace, emitting directly	4.35
	Pulverized coal furnace, integration of wet dust removal and desulfurization	4.162	Pulverized coal furnace, emitting directly	5.04
	CFB boiler, emitting directly	3.672	CFB boiler, emitting directly	3.63
	CFB boiler, integration of wet dust removal and desulfurization	1.102		

Unit category	SO_2 emission factor		NO_x emission factor	
	Technology	kg/t	Technology	kg/t
Coal gangue-fired	In-furnace desulphurization	2.84	Emitting directly	0.95
Diesel-fired	Emitting directly	4.21	Low-nitrogen combustion	3.41
Municipal solid waste-incinerated mixed with coal	Semi-dry absorber	0.526	Low-nitrogen combustion	1.52
Biomass	Emitting directly	1.7	Emitting directly	1.02
Unit category	SO_2 emission factor		NO_x emission factor	
	Technology	g/m^3	Technology	g/m^3
Natural gas-fired	Emitting directly	0.0707	Low-nitrogen combustion	1.66
Coal gas-fired	Emitting directly	0.4	Emitting directly	0.86

Note: a) CFB = circulating fluidized bed; b) SNCR = selective non-catalytic reduction. Some emission factors are calculated by parameters in Supplementary Table 11. For the units without available desulfurization technology information, the average SO_2 emission factor of corresponding capacity-level units are valued for them.

Source: Ministry of Environmental Protection of China (Now renamed as Ministry of Ecology and Environment of China). Manual of Industrial Pollution Source Coefficients (Revised in 2010). 2010.

Supplementary Table 11. Volatile content and sulfur content of coal

Capacity level of coal-fired unit	Coal volatile content (%)	Coal sulfur content (%)
≥600MW	26.58	1.19
300MW	26.32	0.926
100-225MW	25.11	0.816

Source:

China Electricity Council. Basic Information Tables for Thermal Power Generating Units at Capacity Level of 100-225 MW, 300 MW and ≥600 MW. 2014. (The values in the table are taking the average of each capacity-level units.)

Supplementary Table 12. Summary of the GHG mitigation measures and their clients

Scenario	Measure	Client					Additional requirement	Detailed description
		Fuel shift	Unit capacity	Energy output	Technology	Total retrofitted capacity		
Single-measure	M1	Coal to NG	≤120 MW	Electricity	PC	36,949 t/h (boiler)	NG consumption quota	Sup. Note 4.1
	M2	Coal to MSW	3~30 MW	CHP	EC or BP	74,661 t/h (boiler)	MSW consumption quota and unit vintage	Sup. Note 4.2
	M3	Coal (unchanged)	≤200 MW	Electricity or CHP	PC or EC	98,241 MW (turbine)	-	Sup. Note 4.3
	M4		<300 MW		PC, EC or BP	65,242 MW (unit)	Geographic proximity and low energy efficiency	Sup. Note 4.4
	M5	Coal to NG	≤200 MW	Electricity	PC	14,889 MW (unit)	Geographic proximity	Sup. Note 4.5
Integrated	M1&M3	Coal to NG	≤120 MW	Electricity	PC	12,552 t/h (boiler) + 5,919 MW (turbine)	NG consumption quota	Sup. Note 4.6
	M2	Coal to MSW	3~30 MW	CHP	EC or BP	25,952 t/h (boiler)	MSW consumption quota and unit vintage	
	M2&M3				EC	44,269 t/h (boiler) + 4,634 MW (turbine)		
	M3	Coal (unchanged)	≤200 MW	Electricity or CHP	PC or EC	60,352 MW (turbine)	-	
	M4		<300 MW		PC, EC or BP	24,780 MW (unit)	Geographic proximity and low energy efficiency	
	M5	Coal to NG	≤200 MW	Electricity	PC	14,779 MW (unit)	Geographic proximity	

Note: CHP = Combined heat and power; PC = Pure condensing; EC = Extraction condensing; BP = Back pressure; "&" refers to the combination of coupled measures.

Supplementary Table 13. Indicators of electricity benefit variation accounting

Scenario	Parameter/Variable	Value	Unit
All	Coal-based electricity price	0.419[a]	CNY/kWh
	NG-based electricity price	0.758[a]	
	MSW-based electricity price	0.65[b]	
M1	Cumulative added NG-based electricity during the remaining lifetime of the stocks / Cumulative reduced coal-based electricity	562,783,554	MWh
M2	Cumulative added MSW-based electricity / Cumulative reduced coal-based electricity	760,176,969	
M5	Cumulative added NG-based electricity / Cumulative reduced coal-based electricity	1,971,055,621	
Integrated	Cumulative reduced coal-based electricity	3,052,217,916	
	Cumulative added NG-based electricity	2,292,040,946	
	Cumulative added MSW-based electricity	760,176,969	

Source:

a) National Energy Administration of China. Price report on electricity industry in China (2013-2014). 2015. http://zfxxgk.nea.gov.cn/auto92/201509/t20150902_1959.htm.

b) National Development and Reform Commission of China. Municipal solid waste-based electricity price. 2012. http://www.ndrc.gov.cn/zcfb/zcfbtz/201204/t20120410_472395.html.

Supplementary Table 14. Technology structure of energy infrastructure in the parks

Technology	Capacity		Quantity	
	Value (MW)	Share (%)	Value	Share (%)
Pure condensing (PC)	247,251	48.1	1,471	32.4
Extraction condensing (EC)	209,945	40.8	2,157	47.5
Back pressure (BP)	7,364	1.4	475	10.5
Natural gas combined cycle (NGCC)	40,614	7.9	177	3.9
Coal gas combined cycle (CGCC)	1,215	0.2	7	0.2
Integrated gasification combined cycle (IGCC)	265	0.1	1	0.0
Heat generating only (no turbine)	7,878	1.5	254	5.6
Total in-use units	514,533	100	4,542	100

Note: There are 164 units (9,013 MW) retired among all the 4706 units (523,546 MW).

Supplementary Table 15. Efficiencies of electricity supply for different capacity-level units for M4

Unit capacity (MW)	Electricity supply efficiency (g standard coal equivalent/kWh)	Electricity supply efficiency (%)
≥1000	287	42.8
600≤ ＜1000	309	39.8
300≤ ＜600	305	40.3
200≤ ＜300	324	37.9
100≤ ＜200	327	37.6
6≤ ＜100	355	34.6

Source: China Electricity Council. Statistical Data of Chinese Electricity Industry (2015). *China Electricity Council: Beijing*. 2016.

Supplementary Table 16. Indicators of equipment cost accounting

Scenario	Parameter/Variable	Value	Unit
All	Unit cost of retrofitting coal-fired boilers to NG-fired boilers	176,683[a]	CNY/(t/h)
	Unit cost of newly built MSW-driven boilers	798,851[b]	CNY/(t/h)
	Unit cost of retrofitting extraction-condensing/pure-condensing turbines to back-pressure turbines	73,000[c]	CNY/MW
	Unit cost of newly built 350 MW-level coal-fired units	3,281,000[d]	CNY/MW
	Unit cost of newly built 660 MW-level coal-fired units	2,912,000[d]	CNY/MW
	Unit cost of newly built 1000 MW-level coal-fired units	2,904,000[d]	CNY/MW
	Unit cost of newly built 180 MW-level NGCC units	2,874,000[d]	CNY/MW
	Unit cost of newly built 300 MW-level NGCC units	2,407,000[d]	CNY/MW
M1	Added annual NG consumption	445,364,391	GJ
	Total capacity of coal-fired boilers retrofitted to NG-fired boilers	36,949	t/h
M2	Added annual MSW consumption	1,027,781,836	GJ
	Added annual coal consumption to mix with MSW	114,197,982	GJ
	Total capacity of newly built MSW incinerators	74,661	t/h
M3	Total capacity of extraction-condensing and pure-condensing turbines retrofitted to back-pressure turbines	98,421	MW
M4	Total capacity of original units to be replaced	65,242	MW
	Total capacity of newly built 350 MW-level coal-fired units	22,820	MW
	Total capacity of newly built 660 MW-level coal-fired units	39,930	MW
	Total capacity of newly built 1000 MW-level coal-fired units	13,060	MW
M5	Total capacity of original units to be replaced	14,889	MW
	Total capacity of newly built 180 MW-level NGCC units	9,780	MW
	Total capacity of newly built 300 MW-level NGCC units	7,200	MW
Integrated	Total capacity of coal-fired boilers retrofitted to NG-fired boilers	12,552	t/h
	Total capacity of newly built MSW incinerators	70,220	t/h
	Total capacity of extraction-condensing and pure-condensing turbines retrofitted to back-pressure turbines	70,905	MW
	Total capacity of original units to be replaced by large-capacity coal-fired units	24,780	MW
	Total capacity of newly built 350 MW-level coal-fired units	7,860	MW
	Total capacity of newly built 660 MW-level coal-fired units	17,580	MW
	Total capacity of newly built 1000 MW-level coal-fired units	4,060	MW
	Total capacity of original units to be replaced by large-capacity NGCC units	14,779	MW
	Total capacity of newly built 180 MW-level NGCC units	8,100	MW
	Total capacity of newly built 300 MW-level NGCC units	8,700	MW

Note:

1 t/h=0.65 MW[16], 1 MWh=3.6 GJ. The national average annual working time for thermal power plants in 2014 was 4,739 hours/a[20]. The added annual NG consumption in the M1 scenario and the added annual MSW consumption in the M2 scenario were determined first (see Supplementary Note 4); then,

the capacity (t/h) of boilers to be retrofitted or newly built was derived as *Added annual fuel consumption (GJ)* × *Boiler efficiency (BE) ÷3.6 ÷4,739 ÷0.65*.

Source:

a) Tang, G., Yang, Y., Chang, Z. Economic comparison of gas-fired boiler with new coal-fired boiler (in Chinese). *Large Scale Nitrogenous Fertilizer Industry*. 2014**,** 37(6), 408-412.

b) Chinese Nonferrous Engineering Design and Research Institute of China. Feasibility report of Huilian combined heat and power project in Wuxi City (in Chinese). 2004. https://wenku.baidu.com/view/ddc48fdbb1717fd5360cba1aa8114431b90d8ee4.html.

c) Chang, X., Wang, F. Feasibility of retrofitting extraction-condensing turbine to back-pressure turbine (in Chinese). *Chemical Fertilizer Design*. 2014, 52(4), 35-37.

d) Electric Power Planning and Design Institute of China. Cost reference for thermal power engineering design 2015 (in Chinese). *China Electricity Council: Beijing*. 2016.

Supplementary Table 17. Indicators of fuel cost variation accounting

Scenario	Parameter/Variable	Value	Unit
All	Coal price	1,082[a]	CNY/tce
	NG price	1,887[b]	
	MSW subsidy	368[c]	
	Diesel price	4,366[d]	
	Blast furnace gas price	1,555[e]	
	Coke oven gas price	1,400[e]	
	Coal gangue price	210[f]	
	Biomass price	1,700[g]	
	Biogas price	1,401[h]	
M1	Cumulative added NG consumption during the remaining lifetime of the stocks	6,359,921,780	GJ
	Cumulative reduced coal consumption	6,883,680,044	
M2	Cumulative added MSW consumption	23,341,061,961	
	Cumulative net reduced coal consumption	19,527,162,948	
M3	Cumulative reduced coal consumption	17,474,564,123	
	Cumulative reduced NG consumption	175,008,433	
	Cumulative reduced diesel consumption	71,483,490	
	Cumulative reduced blast furnace gas consumption	730,707,859	
	Cumulative reduced coke oven gas consumption	128,417,935	
	Cumulative reduced coal gangue consumption	1,816,639,137	
	Cumulative reduced biomass consumption	4,968,683,587	
	Cumulative reduced biogas consumption	3,267,532	
M4	Cumulative reduced coal consumption	11,658,912,288	
M5	Cumulative added NG consumption	14,743,756,761	
	Cumulative reduced coal consumption	20,559,645,693	
Integrated	Cumulative reduced coal consumption	57,834,456,424	
	Cumulative added NG consumption	16,789,178,381	
	Cumulative added MSW consumption	21,977,994,492	
	Cumulative reduced diesel consumption	71,483,490	
	Cumulative reduced blast furnace gas consumption	730,707,859	
	Cumulative reduced coke oven gas consumption	128,417,935	
	Cumulative reduced coal gangue consumption	1,816,639,137	
	Cumulative reduced biomass consumption	757,530,287	
	Cumulative reduced biogas consumption	3,267,532	

Note: 1 tce (tonne of standard coal equivalent) = 29.27 GJ.

Source:

a) Steam prices of heat supply enterprises in Shanghai. http://fgw.sh.gov.cn/gk/cxgk/14449.htm.

b) Industrial natural gas prices in Beijing. http://www.bjpc.gov.cn/zwxx/tztg/201511/t9778184.htm.

c) Establishing control and accounting platform of municipal solid waste treatment in Beijing. http://law.wkinfo.com.cn/legislation/detail/MTAwMDEzOTAwNjY%3D.

d) Diesel price. https://zhidao.baidu.com/question/649478163904292685.

e) Coal gas prices. https://www.zybang.com/question/d574ec65c21c9d45c9f1c7a69484062d.html.

f) Coal gangue market prices. https://detail.1688.com/offer/575093354600.html?spm=a261b.8768596.0.0.3a991b5cBpNV5W.

g) Biomass market price. http://p4pdetail.hc360.com/p4pdetail/aladindex.html?confr=8&key=%25C9%25FA%25CE%25EF%25D6%25CA%25BF%25C5%25C1%25A3%25B3%25A7%25BC%25D2&bcid=684688567.

h) Biogas market price. https://zhidao.baidu.com/question/96902446.html.

Supplementary Table 18. Material consumption factors for retrofitting or constructing units

Item	Unit material consumption				
	Unit	Concrete	Steel	Iron	Aluminum
Constructing large-capacity coal-fired unit[a]	kg/MW	158,758	50,721	619	419
Constructing large-capacity NGCC unit[b]	kg/MW	97,749	31,030	408	204
Retrofitting coal-fired boiler to NG-fired[c]	kg/(t/h)	-	75.5	-	-
Constructing MSW incinerator[d]	kg/(t/h)	-	1591.8	-	-
Upgrading extraction-condensing / pure-condensing turbine to back-pressure[e]	kg/MW	-	5052.7	-	-

Source:

a) U.S. National Renewable Energy Laboratory. Life Cycle Assessment of Coal-fired Power Production. 1999. https://www.nrel.gov/docs/fy99osti/25119.pdf.

b) U.S. National Renewable Energy Laboratory. Life Cycle Assessment of a Natural Gas Combined Cycle Power Generation System. 2000. https://digital.library.unt.edu/ark:/67531/metadc724566/?q=life%20cycle%20assessment%20.

c) Retrofitting coal-fired steam boiler to natural gas-fired steam boiler. https://wenku.baidu.com/view/d4944dda4afe04a1b171de0a.html.

d) Ao, Y., Cui, W. Installation technology of municipal solid waste incinerator (in Chinese). *Installation*. 2008, 5, 23-25.

e) Li, F. Retrofitting 3000 kW extraction-condensing units to back-pressure units (in Chinese). *District Heating*. 2015, 6, 85-89.

Supplementary Table 19. Indicators of material consumption

Scenario	Parameter/Variable	Value	Unit
M1	Total capacity of coal-fired boilers retrofitted to NG-fired boilers	36,949	t/h
M2	Total capacity of newly built MSW incinerators	74,661	t/h
M3	Total capacity of extraction-condensing and pure-condensing turbines retrofitted to back-pressure turbines	98,241	MW
M4	Total capacity of newly built large-capacity coal-fired units	75,810	MW
M5	Total capacity of newly built large-capacity NGCC units	16,980	MW
Integrated	Total capacity of coal-fired boilers retrofitted to NG-fired	12,552	t/h
	Total capacity of newly built MSW incinerators	70,220	t/h
	Total capacity of extraction-condensing and pure-condensing turbines retrofitted to back-pressure turbines	70,905	MW
	Total capacity of newly built large-capacity coal-fired units	29,500	MW
	Total capacity of newly built large-capacity NGCC units	16,800	MW

Supplementary Table 20. Life-cycle factors for the upstream processes of fuels, water, and materials

Upstream process		Life-cycle GHG emission factor		Life-cycle freshwater consumption factor		Life-cycle SO_2 emission factor		Life-cycle NO_x emission factor	
		Value	Unit	Value	Unit	Value	Unit	Value	Unit
Fuel	Coal	0.179	kg/kg	0.349	m^3/t	2.38E-04	kg/kg	3.62E-04	kg/kg
	Natural gas	0.278	kg/m^3	0.801	kg/m^3	5.30E-04	kg/m^3	3.71E-04	kg/m^3
	Diesel	0.801	kg/kg	7.328	m^3/t	2.67E-03	kg/kg	1.71E-03	kg/kg
	Blast furnace gas	0.234	kg/m^3	2.306	kg/m^3	5.63E-04	kg/m^3	5.02E-04	kg/m^3
	Coke oven gas	0.298	kg/m^3	2.527	kg/m^3	8.24E-04	kg/m^3	6.67E-04	kg/m^3
Water	Industrial tap water	0.190	kg/m^3	1.02	m^3/m^3	5.82E-04	kg/ m^3	4.86E-04	kg/ m^3
Material	Concrete	0.147	kg/kg	0.310	m^3/t	1.20E-04	kg/kg	3.75E-04	kg/kg
	Steel	2.314	kg/kg	16.55	m^3/t	5.47E-03	kg/kg	4.60E-03	kg/kg
	Iron	1.819	kg/kg	14.00	m^3/t	4.67E-03	kg/kg	3.84E-03	kg/kg
	Aluminum	21.66	kg/kg	99.18	m^3/t	6.84E-02	kg/kg	5.23E-02	kg/kg

Note: The factors are cited from a professional LCA database, Chinese Life Cycle Database (CLCD)[23]. The CLCD is a localized database for China, and has been increasingly employed in the studies related to Chinese issues[24]. The upstream processes include production and transportation/transmission.

Supplementary Table 21. Direct fuel, water and material consumption variations in M1-M5 and integrated scenarios

Scenario	Parameter/Variable	Value	Unit
M1	Cumulative added NG consumption during the remaining lifetime of the stocks	6,359,921,780	GJ
	Cumulative reduced coal consumption	6,883,680,044	GJ
	Cumulative added freshwater consumption	158,795,607	m^3
	Cumulative added steel consumption	2,788	t
M2	Cumulative net reduced coal consumption	19,527,162,948	GJ
	Cumulative added freshwater consumption	1,917,233,586	m^3
	Cumulative added steel consumption	118,845	t
M3	Cumulative reduced coal consumption	17,474,564,123	GJ
	Cumulative reduced NG consumption	175,008,433	GJ
	Cumulative reduced diesel consumption	71,483,490	GJ
	Cumulative reduced blast furnace gas consumption	730,707,859	GJ
	Cumulative reduced coke oven gas consumption	128,417,935	GJ
	Cumulative reduced freshwater consumption	19,085,748,472	m^3
	Cumulative added steel consumption	496,377	t
M4	Cumulative reduced coal consumption	11,658,912,288	GJ
	Cumulative reduced freshwater consumption	8,184,385,056	m^3
	Cumulative added concrete consumption	12,035,444	t
	Cumulative added steel consumption	3,845,159	t
	Cumulative added iron consumption	46,926	t
	Cumulative added aluminum consumption	31,764	t
M5	Cumulative added NG consumption	14,743,756,761	GJ
	Cumulative reduced coal consumption	20,559,645,693	GJ
	Cumulative reduced freshwater consumption	2,428,920,050	m^3
	Cumulative added concrete consumption	1,659,778	t
	Cumulative added steel consumption	526,889	t
	Cumulative added iron consumption	6,928	t
	Cumulative added aluminum consumption	3,464	t
Integrated	Cumulative reduced coal consumption	57,834,456,424	GJ
	Cumulative added NG consumption	16,789,178,381	GJ
	Cumulative reduced diesel consumption	71,483,490	GJ
	Cumulative reduced blast furnace gas consumption	730,707,859	GJ
	Cumulative reduced coke oven gas consumption	128,417,935	GJ
	Cumulative reduced freshwater consumption	23,283,824,323	m^3
	Cumulative added concrete consumption	6,325,544	t
	Cumulative added steel consumption	2,488,558	t
	Cumulative added iron consumption	25,115	t
	Cumulative added aluminum consumption	15,788	t

Supplementary Notes

Supplementary Note 1 Scenario timeline

Based on related national guidelines, all the stocks of energy infrastructure in the parks were set to have a 30-year service lifetime[1]. M1-M5 were only applicable to their tailored clients, and the stocks appropriate for retrofitting were matched with the most suitable mitigation measure among M1-M5. The measure-unit matchmaking is detailed in Supplementary Note 4. Supplementary Figure 5 shows the timeline of unit lifetime and the effective working period of different measures. In the baseline scenario, all stocks will retire normally, while in the M1-M5 and integrated scenarios, an overnight retrofitting at T^0 was assumed before the stocks retire. T^0 represents the time when each measure begins for the appropriate clients, which was 2015 in this study. Therefore, the effective working period of M1, M2, and M3 was [T^0, v_{ij} +30], while that of M4 and M5 was [T^0, T^0 + 30]. All the measures were incorporated in the integrated scenario, and "&" refers to the combination of coupled measures. All the variables and parameters of the vintage stock model were defined as Supplementary Table 5, and the parameters were valued as Supplementary Tables 6-11.

Supplementary Note 2 Energy efficiency assessment of energy infrastructure

The energy efficiency assessment of the in-use energy infrastructure stocks in ~100 Chinese eco-industrial parks can be found in previous work[2]. In this study, we used a similar method to assess the efficiencies of all the in-use energy infrastructure stocks in the 1,604 parks. The method is presented as follows.

Energy infrastructure basically generates electricity, heat or both. There were 2,127 energy infrastructure plants in our database. The plants generally included several units, and plant-level technical data were available. Considering the working mechanism of the combined heat and power (CHP) system (see Supplementary Figure 6), we used the steam from boiler (*SFB*) metric to incorporate the steam consumed for both electricity generation and industrial processes. The *SFB* refers to the steam that is generated from boilers and then passes into turbines. The turbines allocate the *SFB* energy to generate electricity and heat, which fluctuates with the steam loading of users. Thus, the energy efficiency (*EE*) can be derived by dividing the total fuel input with the total energy output, as

shown in equation 1. The electricity output (*EO*) and heat output (*HO*) were calculated by equations 2 and 3, respectively.

For further assessing the performance of energy infrastructure, determining the effective electrical efficiency (*EEE*) is more useful than measuring the overall *EE* for fuel-saving evaluations. According to CHP guidelines[3], the *EEE* is calculated assuming that the *HO* from CHP systems would otherwise be generated by industrial boilers driven by the same fuel. Then, the total fuel input can be divided into the fuel for *EO* and the fuel for *HO*; see equations 4 and 5, respectively. For CHP plants, equation 1 can be transformed by dividing the numerator and denominator by the *EO*, as in equation 6. For electricity-generating plants, the *HO* and fuel for heat *(FFH)* are both zero, so *EE = EEE*. For heat-generating plants, the *EO* and fuel for electricity (*FFE*) are zero, and the *HO* is equal to *SFB* value, so *EE=boiler efficiency (BE)*. Thus, the *EE* of energy infrastructure can be summarized, as shown in equation 6. For some data-unavailable units, their *EEE, BE*, and heat-to-electric ratio (*H/E*) take the default values of data-available units in our database (see Supplementary Tables 1-3).

$$Energy\ Efficiency\ (EE)=\frac{Electricity\ Output\ (EO)+Heat\ Output\ (HO)}{Fuel\ for\ Electricity\ (FFE)+Fuel\ for\ Heat\ (FFH)} \tag{1}$$

$$EO_i=\sum_j (Cap_{ij}\times WorkTime_i\times\left(1\text{-}SelfUseRate_i\right)) \tag{2}$$

$$HO_i=\sum_j (Cap_{ij}\times WorkTime_i\times(H/E)_i) \tag{3}$$

$$BE_i=SFB_i/(FFE_i+FFH_i)=HO_i/FFH_i \tag{4}$$

$$EEE_i=EO_i/FFE_i \tag{5}$$

$$EE_i=\begin{cases}\frac{1+(H/E)_i}{1/EEE_i+(H/E)_i/BE_i} & CHP\ plant\\ EEE_i & electricity\text{-}generating\ plant\\ BE_i & heat\text{-}generating\ plant\end{cases} \tag{6}$$

Supplementary Note 3 Direct environmental impact accounting in 2014 and the baseline scenario

In 2014, the GHG emissions, freshwater consumption, SO_2 emissions, and NO_x emissions of all the energy infrastructure stocks in the 1,604 parks were accounted by equations 7-10. The national GHG emissions, national industrial freshwater consumption, national SO_2 emissions and national NO_x emissions were 13.12 Gt CO_2 eq., 74.11 Gm^3, 2.35 Mt, and 3.07 Mt, respectively[4, 5, 6]. Thus, the

percentages of the stocks to the whole country were then calculated. The GHG emissions considered in this study included CO_2, CH_4, and N_2O, which were converted to CO_2 equivalent according to the 100-year global warming potential (see Supplementary Table 7).

$$GHGEmi^{2014} = \sum_{i=1}^{2127} \sum_{j=1}^{Num_i} ((EO_{ij}+HO_{ij}) \div EE_i^{BA} \times GHGFac(Fuel_{ij})) \tag{7}$$

$$WaterCon^{2014} = \sum_{i=1}^{2127} \sum_{j=1}^{Num_i} (EO_{ij}/(1\text{-}SelfUseRate_i) \times WatFacEle(CoolTech_{ij}, Fuel_{ij}, Cap_{ij}) + HO_{ij} \times WatFacHeat(Prov_i)) \tag{8}$$

$$SO2Emi^{2014} = \sum_{i=1}^{2127} \sum_{j=1}^{Num_i} ((EO_{ij}+HO_{ij}) \div EE_i^{BA} \times SO2Fac(Fuel_{ij})) \tag{9}$$

$$NOxEmi^{2014} = \sum_{i=1}^{2127} \sum_{j=1}^{Num_i} ((EO_{ij}+HO_{ij}) \div EE_i^{BA} \times NOxFac(Fuel_{ij})) \tag{10}$$

In the baseline scenario, the energy infrastructure stocks work until they retire normally, and the technical attributes of the stocks (i.e., fuel and technology) during their remaining lifetime period [T^0, v_{ij}+30] remain the same as those in 2014. All the units had data on individual capacity, fuel type, turbine technology, cooling technology, and vintage, but unit-level operational data, such as annual working time and energy efficiency, were unavailable for some units. Thus, plant-level operational data were used for the units belonging to each plant. Thus, the environmental impacts of the stocks during their remaining lifetimes were accounted in equations 11-14.

$$GHGEmi^{BA} = \sum_{i=1}^{2127} \sum_{j=1}^{Num_i} ((EO_{ij}+HO_{ij}) \div EE_i^{BA} \times RemLife_{ij} \times GHGFac(Fuel_{ij})) \tag{11}$$

$$WaterCon^{BA} = \sum_{i=1}^{2127} \sum_{j=1}^{Num_i} (EO_{ij}/(1\text{-}SelfUseRate_i) \times WatFacEle(CoolTech_{ij}, Fuel_{ij}, Cap_{ij}) + HO_{ij} \times WatFacHeat(Prov_i)) \times RemLife_{ij}) \tag{12}$$

$$SO2Emi^{BA} = \sum_{i=1}^{2127} \sum_{j=1}^{Num_i} ((EO_{ij}+HO_{ij}) \div EE_i^{BA} \times RemLife_{ij} \times SO2Fac(Fuel_{ij}, Cap_{ij})) \tag{13}$$

$$NOxEmi^{BA} = \sum_{i=1}^{2127} \sum_{j=1}^{Num_i} ((EO_{ij}+HO_{ij}) \div EE_i^{BA} \times RemLife_{ij} \times NOxFac(Fuel_{ij}, Cap_{ij})) \tag{14}$$

Supplementary Note 4 Direct environmental benefits in the M1-M5 and integrated scenarios

Measure-unit matchmaking considers the attributes of units (such as capacity, fuel, technology, and efficiency) and additional requirements (such as vintage, geographic proximity, and fuel consumption

quota). The GHG mitigation measures and their clients are summarized in Supplementary Table 12, and detailed descriptions are presented as follows.

M1 scenario: retrofitting coal-fired boilers to natural gas-fired boilers

The energy infrastructure stocks in the parks are heavily dependent on coal, as 87% of the total capacity is coal-fired units. Natural gas (NG) is a strategic energy option for China and should be optimally used under limited quotas. Large-capacity (300 MW or more) coal-fired units are highly efficient[7], and have been retrofitted to ultralow emission units that can meet the emission standard of NG-fired units after intensified dust removal, desulfurization, and denitrification[8]. Additionally, NG-based electricity has a higher feed-in tariff than coal-based electricity[9] (see Supplementary Table 13), which will lead to more revenue from selling electricity. Thus, in the M1 scenario, coal-fired electricity units with a capacity of no more than 120 MW are retrofitted from coal-fired boilers to NG-fired boilers. The new boilers will generate the same quantity of steam as the original boilers.

The added NG consumption in the M1 scenario will be 11.4 Gm^3 per year. The NG consumption for electricity and heat generation in China increased from 21.1 Gm^3 in 2010 to 37.4 Gm^3 in 2015[10, 11]. The effective period of M1 is averagely from 2015-2029 (M1 clients have an average remaining lifetime of 15 years), and therefore, it is reasonable that the added annual NG associated with M1 should not exceed the growth from 2011-2015 (16 Gm^3). In addition, water consumption and air pollutant emissions will also change along with the retrofit. Such a carbon-water-pollutant nexus should be considered. The GHG mitigation potentials, freshwater savings, and SO_2 and NO_x emission reductions were quantified in equations 15-18. “PC” refers to a unit having a pure-condensing turbine, meaning that the energy infrastructure is an electricity-generating plant.

$$GHGMit_D^{M1} = \sum_{i=1}^{2127} \sum_{j=1}^{Num_i} (GHGEmi_{ij}^{BA} \times (1 - \frac{BE_{ij}}{BE^{NG}} \times \frac{GHGFac(NG)}{GHGFac(Coal)}))|_{Fuel_{ij}=Coal \text{ and } Cap_{ij} \leq 120 \text{ MW and } TurTech_{ij}=PC} \quad (15)$$

$$WatSav_D^{M1} = \sum_{i=1}^{2127} \sum_{j=1}^{Num_i} (EO_{ij}/\left(1\text{-}SelfUseRate_i\right) \times RemLife_{ij} \times (WatFacEle\left(CoolTech_{ij}, Coal, Cap_{ij}\right) - WatFacEle\left(CoolTech_{ij}, NG\right)))|_{Fuel_{ij}=Coal \text{ and } Cap_{ij} \leq 120 \text{ MW and } TurTech_{ij}=PC} \quad (16)$$

$$SO2Red_D^{M1} = \sum_{i=1}^{2127} \sum_{j=1}^{Num_i} (SO2Emi_{ij}^{BA} - (EO_{ij} + HO_{ij}) \div EE_i^{BA} \times RemLife_{ij} \times \frac{BE_{ij}}{BE^{NG}}$$

$$\times SO2Fac(NG))|_{Fuel_{ij}=Coal\ and\ Cap_{ij}\leq 120\ MW\ and\ TurTech_{ij}=PC} \tag{17}$$

$$NOxRed_{D}^{M1}=\sum_{i=1}^{2127}\sum_{j=1}^{Num_i}(NOxEmi_{ij}^{BA}-(EO_{ij}+HO_{ij})\div EE_{i}^{BA}\times RemLife_{ij}\times\frac{BE_{ij}}{BE^{NG}}$$

$$\times NOxFac(NG))|_{Fuel_{ij}=Coal\ and\ Cap_{ij}\leq 120\ MW\ and\ TurTech_{ij}=PC} \tag{18}$$

M2 scenario: replacing coal-fired boilers with MSW incinerators

Municipal solid waste (MSW) is mostly treated by landfilling in China, which leads to land scarcity and heavy pollution[12]. As a result, MSW incineration (MSWI) has been promoted by the Chinese government in the 13th Five-year Plan[13]. Moreover, MSWI is widely practiced in Chinese industrial parks with a total capacity of 2.4 GW, accounting for 55% of the total MSWI capacity in China[14]. A previous study showed that MSWI facilities have an individual capacity of 3~30 MW, and that improved environmental performance can be achieved by using MSWI units to generate both electricity and heat[14]. Besides, the lifetime of MSWI facilities is at least 20 years according to national design guidelines[15]. Thus, in the M2 scenario, MSW incinerators are used to partly replace the boilers of coal-fired CHP units.

The MSWI capacity in 2014 was 4.3 GW in China, and the national target in 2020 is 7.5 GW[13]. Along with this annual growth rate, the MSWI capacity in 2030 will reach 18.9 GW. We assume that the ratio of MSWI capacity in the industrial parks to that of the whole country will remain at approximately 50%, and therefore, the in-use MSWI facilities in the parks by 2030 will be 9.4 GW in China. Thus, the added MSWI capacity will be approximately 7 GW from 2015-2030, which is the total capacity of the retrofitted coal-fired CHP units in the M2 scenario. Therefore, the client units of M2 were determined by the following steps. (1) Coal-fired CHP units with a capacity of 3 ~ 30 MW and a remaining lifetime of 20 years or longer were defined as the units to be retrofitted. (2) Then, the GHG emission mitigation potentials of the units were calculated using equation 19. The units to be retrofitted were ranked from largest to smallest by GHG mitigation potential per MW. (3) The preferential units in the list with an aggregated capacity of 7 GW were identified as the M2 clients, and their boilers were targeted to be replaced by MSW incinerators. Generally, MSW needs to be mixed with coal for stable combustion, and the mix rate of coal to the total fuel input is referred to Supplementary Table 1. Thus, the environmental benefits of M2 are quantified, as calculated by equations 19-22.

$$GHGMit_D^{M2}=\sum_{i=1}^{2127}\sum_{j=1}^{Num_i}(GHGEmi_{ij}^{BA}\times(1-\frac{BE_{ij}}{BE^{MSW}}\times\frac{(1-MixRate)\times GHGFac(MSW)+MixRate\times GHGFac(Coal)}{GHGFac(Coal)}))|_{M2\ client} \tag{19}$$

$$WatSav_D^{M2}=\sum_{i=1}^{2127}\sum_{j=1}^{Num_i}(EO_{ij}/\left(1\text{-}SelfUseRate_i\right)\times RemLife_{ij}\times(WatFacEle\left(CoolTech_{ij},Coal,Cap_{ij}\right)-WatFacEle\left(CoolTech_{ij},MSW\right))|_{M2\ client} \tag{20}$$

$$SO2Red_D^{M2}=\sum_{i=1}^{2127}\sum_{j=1}^{Num_i}(SO2Emi_{ij}^{BS}-(EO_{ij}+HO_{ij})\div EE_i^{BA}\times RemLife_{ij}\times\frac{BE_{ij}}{BE^{MSW}}\times SO2Fac(MSW))|_{M2\ client} \tag{21}$$

$$NOxRed_D^{M2}=\sum_{i=1}^{2127}\sum_{j=1}^{Num_i}(NOxEmi_{ij}^{BS}-(EO_{ij}+HO_{ij})\div EE_i^{BA}\times RemLife_{ij}\times\frac{BE_{ij}}{BE^{MSW}}\times NOxFac(NG))|_{M2\ client} \tag{22}$$

M3 scenario: retrofitting extraction-condensing or pure-condensing turbines to back-pressure turbines

The M3 scenario is related to CHP technology upgrades. Recent national energy strategies have requested that extraction-condensing or pure-condensing units with a capacity of 200 MW or less should be upgraded to back-pressure units by retrofitting their turbines[7]. Back-pressure units work under the principle of preferentially generating heat, while electricity generation is supplementary. These units generally have a higher energy efficiency than other CHP units[3]. Among all the stocks, extraction-condensing and pure-condensing units accounted for 48% and 41% of the total capacity, respectively; back-pressure units only take a share of 1.4% by 2014 (see Supplementary Table 14). The average EEE of back-pressure units is 12.4% and 19.2% higher than that of extraction-condensing units and pure-condensing units (200 MW or smaller), respectively. Efficiency improvements through turbine retrofit (ΔEEE) were set. Then, the environmental benefits of M3 were quantified by equations 23-26. “EC” refers to an extraction-condensing unit, and “PC” refers to a pure-condensing unit.

$$GHGMit_D^{M3}=\sum_{i=1}^{2127}\sum_{j=1}^{Num_i}(GHGEmi_{ij}^{BA}\times(1-EE_{ij}\div\frac{1+(H/E)_i}{1/(EEE_{ij}+\Delta EEE_{ij})+(H/E)_i/BE_{ij}}))|_{(TurTech_{ij}=EC\ or\ PC)\ and\ Cap_{ij}\leq 200MW} \tag{23}$$

$$WatSav_D^{M3}=\sum_{i=1}^{2127}\sum_{j=1}^{Num_i}(EO_{ij}/\left(1\text{-}SelfUseRate_i\right)\times RemLife_{ij}\times(WatFacEle\left(CoolTech_{ij},Fuel_{ij},Cap_{ij}\right)$$

$$-WatFacEle\left(CoolTech^{M3},Fuel_{ij},Cap_{ij}\right))|_{(TurTech_{ij}=EC\ or\ PC)\ and\ Cap_{ij}\leq 200MW} \tag{24}$$

$$SO2Red_D^{M3}=\sum_{i=1}^{2127}\sum_{j=1}^{Num_i}(SO2Emi_{ij}^{BA}\times(1-EE_{ij}$$

$$\div\frac{1+(H/E)_i}{1/(EEE_{ij}+\Delta EEE_{ij})+(H/E)_i/BE_{ij}}))|_{(TurTech_{ij}=EC\ or\ PC)\ and\ Cap_{ij}\leq 200MW} \tag{25}$$

$$NOxRed_D^{M3}=\sum_{i=1}^{2127}\sum_{j=1}^{Num_i}(NOxEmi_{ij}^{BA}\times(1-EE_{ij}$$

$$\div\frac{1+(H/E)_i}{1/(EEE_{ij}+\Delta EEE_{ij})+(H/E)_i/BE_{ij}}))|_{(TurTech_{ij}=EC\ or\ PC)\ and\ Cap_{ij}\leq 200MW} \tag{26}$$

M4 scenario: replacing small-capacity coal-fired units with large-capacity coal-fired units

Among all the energy infrastructure stocks in the parks, 81% of the total quantity had a capacity below 300 MW. According to China's energy strategies, small-capacity coal-fired units are required to be phased out, and newly added coal-fired units should be 300 MW or larger[7]. Some parks have implemented this measure[16], but there are still many parks that need to be improved. In light of technical feasibility and cost effectiveness, the small-capacity coal-fired units to be replaced were identified by the following two criteria: (1) coal-fired units with a capacity below 300 MW and an *EEE* below 40.3% (which is the national average *EEE* of 300 MW coal-fired units[17]); (2) the units meeting criteria (1) within an industrial park have a total capacity of at least 180 MW (the national guideline requests that the total capacity of the small units to be shut down should be at least 60% of the total capacity of the new units[18]). The capacity of alternatives for the M3 clients in an industrial park obeyed the following function: min $\{x$ MW $|\ x \geq$ total capacity of replaced units, $x \in \{300, 330, 350, 600, 630, 660, 1000$, and their linear combinations$\}\}$. The newly established units have an EEE^{M4} based on their capacity levels (see Supplementary Table 15) and the same *BE* as the replaced units. The new units will generate the same energy output as the collection of replaced units and have a serviceable lifetime of 30 years according to the related guidelines[1]. Then, equations 27-30 accounted for the environmental improvements of M4.

$$GHGMit_D^{M4}=\sum_{i=1}^{2127}\sum_{j=1}^{Num_i}(GHGEmi_{ij}^{BA}\times\frac{30}{RemLife_{ij}}\times(1-EE_{ij}\div\frac{1+(H/E)_i}{1/EEE_{ij}^{M4}+(H/E)_i/BE_{ij}})|_{M4\ client} \tag{27}$$

$$WatSav_D^{M4}=\sum_{i=1}^{2127}\sum_{j=1}^{Num_i}(EO_{ij}/\left(1-SelfUseRate_i\right)\times 30\times(WatFacEle\left(CoolTech_{ij},Coal,Cap_{ij}\right)$$

$$-WatFacEle\left(CoolTech_{ij},Coal,Cap_{ij}^{M4}\right))|_{M4\ client} \tag{28}$$

$$SO2Red_D^{M4} = \sum_{i=1}^{2127} \sum_{j=1}^{Num_i} ((SO2Emi_{ij}^{BA} \div RemLife_{ij} - (EO_{ij} + HO_{ij}) \div \frac{1+(H/E)_i}{1/EEE_{ij}^{M4} + (H/E)_i/BE_{ij}} \times SO2Fac\left(Coal, Cap_{ij}^{M4}\right)) \times 30)|_{M4\ client} \quad (29)$$

$$NOxRed_D^{M4} = \sum_{i=1}^{2127} \sum_{j=1}^{Num_i} ((NOxEmi_{ij}^{BA} \div RemLife_{ij} - (EO_{ij} + HO_{ij}) \div \frac{1+(H/E)_i}{1/EEE_{ij}^{M4} + (H/E)_i/BE_{ij}} \times NOxFac\left(Coal, Cap_{ij}^{M4}\right)) \times 30)|_{M4\ client} \quad (30)$$

M5 scenario: replacing small-capacity coal-fired units with large-capacity NGCC units

In the M5 scenario, small-capacity coal-fired units will be replaced with large-capacity NGCC units. Similar to M4, considering technical feasibility and cost effectiveness, the small-capacity coal-fired units to be replaced were identified by the following two criteria: (1) coal-fired electricity units with a capacity no greater than 200 MW; (2) the units meeting criteria (1) within an industrial park have a total capacity of at least 108 MW (the national guideline requires that the total capacity of the small units to be shut down is at least 60% of the total capacity of the new units[18]). The capacity of alternatives for the M5 clients in an industrial park obeyed the following function: min $\{x$ MW $\vert x \geq$ total capacity of replaced units, $x \in$ {180, 300, and their linear combinations}$\}$. The new NGCC units will generate the same energy output as the collection of replaced units and have a serviceable lifetime of 30 years according to the related guidelines[1]. The new NGCC electricity-generating units have an EEE^{M5} of 48.1% (equal to EE^{M5} due to $(H/E)^{M5} = 0$), which is the average *EEE* of the existing NGCC units in the parks according to our database. The added NG consumption in the M5 scenario was 12.6 Gm^3 per year, which is also reasonable, as discussed in the M1 scenario. Then, the environmental impacts of M5 were quantified using equations 31-34.

$$GHGMit_D^{M5} = \sum_{i=1}^{2127} \sum_{j=1}^{Num_i} (GHGEmi_{ij}^{BA} \times \frac{30}{RemLife_{ij}} \times (1 - EE_{ij} \div EE^{M5}))|_{M5\ client} \quad (31)$$

$$WatSav_D^{M5} = \sum_{i=1}^{2127} \sum_{j=1}^{Num_i} (EO_{ij}/\left(1 - SelfUseRate_i\right) \times 30 \times (WatFacEle\left(CoolTech_{ij}, Coal, Cap_{ij}\right) - WatFacEle\left(CoolTech_{ij}, NG\right)))|_{M5\ client} \quad (32)$$

$$SO2Red_D^{M5} = \sum_{i=1}^{2127} \sum_{j=1}^{Num_i} ((SO2Emi_{ij}^{BA} \div RemLife_{ij} - EO_{ij} \div EE^{M5} \times SO2Fac(NG)) \times 30)|_{M5\ client} \quad (33)$$

$$NOxRed_D^{M5} = \sum_{i=1}^{2127} \sum_{j=1}^{Num_i} ((NOxEmi_{ij}^{BA} \div RemLife_{ij} - EO_{ij} \div EE^{M5} \times NOxFac(NG)) \times 30)|_{M5\ client} \quad (34)$$

Integrated scenario

M1-M5 can be integrated in practice. Each unit of the energy infrastructure stocks is mainly made of boilers and turbines. M1 and M2 retrofit boilers, M3 retrofits turbines, and M4 and M5 replace whole units. Based on technical feasibility, M1 and M2 were coupled with M3, called "M1&M3" and "M2&M3", respectively. M1&M3 refers to retrofitting coal-fired boilers to NG-fired boilers, and upgrading extraction-condensing or pure-condensing turbines to back-pressure turbines simultaneously. M2&M3 refers to replacing coal-fired boilers with MSW incinerators, and meanwhile retrofitting extraction-condensing or pure-condensing turbines to back-pressure turbines. Thus, the environmental impacts of these combined measures were presented in equations 35-42.

$$GHGMit_D^{M1\&M3}=\sum_{i=1}^{2127}\sum_{j=1}^{Num_i}(GHGEmi_{ij}^{BA}\text{-}EO_{ij}\times RemLife_{ij}\div(\frac{BE^{NG}}{BE_{ij}}\times\left(EEE_{ij}+\Delta EEE_{ij}\right))\times GHGFac(NG))|_{Fuel_{ij}=Coal\ and\ Cap_{ij}\leq 120\ MW\ and\ TurTech_{ij}=PC} \quad (35)$$

$$WatSav_D^{M1\&M3}=\sum_{i=1}^{2127}\sum_{j=1}^{Num_i}(EO_{ij}/\left(1\text{-}SelfUseRate_i\right)\times RemLife_{ij}\times(WatFacEle\left(CoolTech_{ij},Coal,Cap_{ij}\right)\text{-}WatFacEle\left(CoolTech_{ij}^{M1\&M3},NG\right))|_{Fuel_{ij}=Coal\ and\ Cap_{ij}\leq 120\ MW\ and\ TurTech_{ij}=PC} \quad (36)$$

$$SO2Red_D^{M1\&M3}=\sum_{i=1}^{2127}\sum_{j=1}^{Num_i}(SO2Emi_{ij}^{BA}\text{-}EO_{ij}\times RemLife_{ij}\div(\frac{BE^{NG}}{BE_{ij}}\times\left(EEE_{ij}+\Delta EEE_{ij}\right))\times SO2Fac(NG))|_{Fuel_{ij}=Coal\ and\ Cap_{ij}\leq 120\ MW\ and\ TurTech_{ij}=PC} \quad (37)$$

$$NOxRed_D^{M1\&M3}=\sum_{i=1}^{2127}\sum_{j=1}^{Num_i}(NOxEmi_{ij}^{BA}\text{-}EO_{ij}\times RemLife_{ij}\div(\frac{BE^{NG}}{BE_{ij}}\times\left(EEE_{ij}+\Delta EEE_{ij}\right))\times NOxFac(NG))|_{Fuel_{ij}=Coal\ and\ Cap_{ij}\leq 120\ MW\ and\ TurTech_{ij}=PC} \quad (38)$$

$$GHGMit_D^{M2\&M3}=\sum_{i=1}^{2127}\sum_{j=1}^{Num_i}(GHGEmi_{ij}^{BA}\text{-}(EO_{ij}+HO_{ij})\times RemLife_{ij}\div\frac{1+(H/E)_i}{1/(\frac{BE^{MSW}}{BE_{ij}}\times\left(EEE_{ij}+\Delta EEE_{ij}\right))+(H/E)_i/BE^{MSW}}\times((1\text{-}MixRate)\times GHGFac(MSW)+MixRate\times GHGFac(Coal)))|_{M2\ client\ and\ TurTech_{ij}=EC} \quad (39)$$

$$WatSav_D^{M2\&M3}=\sum_{i=1}^{2127}\sum_{j=1}^{Num_i}(EO_{ij}/\left(1\text{-}SelfUseRate_i\right)\times RemLife_{ij}\times(WatFacEle\left(CoolTech_{ij},Coal,Cap_{ij}\right)\text{-}WatFacEle\left(CoolTech_{ij}^{M2\&M3},MSW\right))|_{M2\ client\ and\ TurTech_{ij}=EC} \quad (40)$$

$$SO2Red_D^{M2\&M3}=\sum_{i=1}^{2127}\sum_{j=1}^{Num_i}(SO2Emi_{ij}^{BA}\text{-}(EO_{ij}+HO_{ij})\times RemLife_{ij}\div\frac{1+(H/E)_i}{1/(\frac{BE^{MSW}}{BE_{ij}}\times\left(EEE_{ij}+\Delta EEE_{ij}\right))+(H/E)_i/BE^{MSW}}$$

$$\times SO2Fac(MSW))|_{M2\ client\ and\ TurTech_{ij}=EC} \quad (41)$$

$$NOxRed_D^{M2\&M3} = \sum_{i=1}^{2127}\sum_{j=1}^{Num_i}(NOxEmi_{ij}^{BA}\text{-}(EO_{ij}+HO_{ij})\times RemLife_{ij}\div\frac{1+(H/E)_i}{1/(\frac{BE^{MSW}}{BE_{ij}}\times(EEE_{ij}+\Delta EEE_{ij}))+(H/E)_i/BE^{MSW}}$$

$$\times NOxFac(MSW))|_{M2\ client\ and\ TurTech_{ij}=EC} \quad (42)$$

Based on the above, in the integrated scenario, each unit of the energy infrastructure stocks will be changed by the measure that can achieve the maximum GHG mitigation potential among its appropriate measures (M1-M5, M1&M3 and M2&M3). Then, the environmental benefits in the integrated scenario could be quantified with equations 43-46. If a unit was not the client of any measure, the GHG mitigation potential and other environmental benefits for applying the measure to this unit would be zero. “X” refers to one of the M1-M5, M1&M3, and M2&M3.

$$GHGMit_D^{IN} = \sum_{i=1}^{2127}\sum_{j=1}^{Num_i} Max\left(GHGMit_{ij}^X\ |X\in\{M1\text{-}M5,\ M1\&M3,\ M2\&M3\}\right) \quad (43)$$

$$WatSav_D^{IN} = \sum_{i=1}^{2127}\sum_{j=1}^{Num_i} WatSav_{ij}^X \quad (44)$$

$$SO2Red_D^{IN} = \sum_{i=1}^{2127}\sum_{j=1}^{Num_i} SO2Red_{ij}^X \quad (45)$$

$$NOxRed_D^{IN} = \sum_{i=1}^{2127}\sum_{j=1}^{Num_i} NOxRed_{ij}^X \quad (46)$$

Supplementary Note 5 Cost-benefit analysis of the M1-M5 and integrated scenarios

The cost-benefit analysis included variations in equipment costs, fuel costs, electricity benefits, and heat benefits, by considering the differences between the GHG mitigation scenarios (the M1-M5 and integrated scenarios) and the baseline scenario. The net cost was quantified with equation 47. The “Δ” symbol refers to the variation in a certain indicator of the GHG mitigation scenarios compared with that of the baseline scenario.

$$\begin{aligned} Net\ Cost &= \Delta Equipment\ Cost+\Delta Fuel\ Cost+\Delta Electricity\ Benefit+\Delta Heat\ Benefit \\ &= \sum(Retrofitted\ or\ constructed\ capacity\times Unit\ cost)+\sum(\Delta Fuel\ input\times Fuel\ price) \\ &\quad +\sum(\Delta Electricity\ output\times Electricity\ price)+\sum(\Delta Heat\ output\times Heat\ price) \end{aligned} \quad (47)$$

The energy outputs of the stocks in the GHG mitigation scenarios kept the same as those in the baseline scenario. However, the energy efficiencies of the client stocks of M1, M3, M4 and M5 were improved, and fuel alternatives were also implemented by M1, M2, and M5. Thus, the fuel inputs and the structure of energy outputs in the GHG mitigation scenarios changed compared with the baseline scenario. Specifically, the GHG mitigation scenarios involved unit retrofitting or construction, shifting coal to NG or MSW, energy efficiency improvements, coal-based electricity reductions, and incremental NG-based or MSW-based electricity.

Moreover, the price indexes of equipment, fuel, and energy fluctuate during the remaining lifetime of the stocks. According to the methods in related studies[19], this study assumed that the inflation rate of the present-value prices for equipment, fuel, and energy in each year were equal to the social discount rate. Therefore, the discount rate could be offset by the inflation rate when summing the present-value costs and benefits of each year during the remaining lifetime of the stocks. The GHG mitigation costs derived by equation 47 were at the constant price of 2015, and the unit cost was the ratio of the total cost to the direct GHG emission reduction for each scenario. The equipment costs, fuel costs, electricity benefits, and heat benefits in the GHG mitigation scenarios are illustrated as follows.

Equipment cost

The total capacity of units to be retrofitted or constructed was determined for each GHG mitigation scenario. Then, based on the unit costs of different facilities, the equipment cost for each scenario were derived as $\sum$ *(Retrofitted or constructed capacity × Unit cost)*. The related indicators are presented in Supplementary Table 16. The equipment costs included the costs of equipment purchase, installation work, construction engineering, and other expenses (such as land occupancy and clearing).

Fuel cost variation

In the GHG mitigation scenarios, the energy outputs of all changed units remained the same as those in the baseline scenario. However, the GHG mitigation measures changed the energy efficiencies of client units, through retrofitting or replacing the units and the fuel alternatives. Thus, the fuel input structure was changed, and the fuel cost variation for each scenario could be quantified by $\sum$ *(ΔFuel input× Fuel price)*. In particular, the subsidies from local government for MSW treatment is

widely implemented in China, so the MSW subsidies were also considered in fuel costs. The indicators of fuel cost variation accounting were presented in Supplementary Table 17.

Electricity benefit variation

The fuel alternatives in M1, M2, M5 and integrated scenarios will lead to changes in the electricity output structure. Therefore, the electricity benefit variation for each scenario was derived as $\sum(\Delta \textit{Electricity output} \times \textit{Electricity price})$. The indicators for accounting the variations in electricity benefits were presented in Supplementary Table 13.

Heat benefit variation

Among the GHG mitigation measures, the fuel shifting-related measures were M1, M2, and M5. M1 and M5 only worked on electricity units, and heat outputs were not changed; M2 retrofits the CHP units, but the heat price from MSW incineration was the same as that from coal firing. Therefore, the heat outputs from each fuel combustion remained unchanged when comparing M1-M5 and integrated scenarios with the baseline scenario. Thus, the heat benefit variation was zero.

Supplementary Note 6 Material consumption in the M1-M5 and integrated scenarios

The GHG mitigation measures will consume materials for retrofitting and constructing units. To assess each measure systematically, the indirect environmental impacts embodied in material consumption should be included. Our model considered four categories of bulk materials: concrete, steel, iron, and aluminum. The production and transportation processes for these four materials contributed the majority of the indirect environmental impacts of unit retrofitting or construction[21, 22]. Each material consumption was derived by equation 48. Through literature review and calculations, the material consumption factors and amounts were listed in Supplementary Tables 18 and 19.

$$\textit{Material Consumption} = \sum(\textit{Retrofitted or constructed capacity} \times \textit{Unit consumption}) \quad (48)$$

Supplementary Note 7 Indirect and total environmental benefits of the M1-M5 and integrated scenarios

Fuel consumption, water consumption, and material consumption of the client stocks will be altered with GHG mitigation measures due to changes in capacity, technology, fuel type, etc. Such an energy-water-material nexus should be considered for a more systematic assessment. Furthermore, from a life-cycle perspective, the GHG mitigation measures will lead to indirect environmental impacts through directly changing fuel consumption, freshwater consumption, and material consumption. The upstream processes (including production and transportation/transmission processes) of fuels, water, and materials were considered in this model, as presented in Supplementary Figure 7.

Thus, the indirect environmental benefits embodied in the direct reduced sections of fuels, water, and materials were presented in equations 49-52. “X” refers to one of the M1-M5 and integrated scenarios.

$$\begin{aligned} GHGMit_I^X = & \sum (Fuel\ Reduction \times GHGFac(Fuel\ Production)) \\ & + Water\ Reduction \times GHGFac(Water\ Production) \\ & + \sum (Material\ Reduction \times GHGFac(Material\ Production)) \end{aligned} \quad (49)$$

$$\begin{aligned} WatSav_I^X = & \sum (Fuel\ Reduction \times WatFac(Fuel\ Production)) \\ & + Water\ Reduction \times WatFac(Water\ Production) \\ & + \sum (Material\ Reduction \times WatFac(Material\ Production)) \end{aligned} \quad (50)$$

$$\begin{aligned} SO2Red_I^X = & \sum (Fuel\ Reduction \times SO2Fac(Fuel\ Production)) \\ & + Water\ Reduction \times SO2Fac(Water\ Production) \\ & + \sum (Material\ Reduction \times SO2Fac(Material\ Production)) \end{aligned} \quad (51)$$

$$\begin{aligned} NOxRed_I^X = & \sum (Fuel\ Reduction \times NOxFac(Fuel\ Production)) \\ & + Water\ Reduction \times NOxFac(Water\ Production) \\ & + \sum (Material\ Reduction \times NOxFac(Material\ Production)) \end{aligned} \quad (52)$$

The life-cycle factors for the upstream processes of fuels, water, and materials were presented in Supplementary Table 20. The direct variations in fuel, water, and material consumption were listed in Supplementary Table 21.

Based on accounting the indirect environmental impacts, the total environmental benefits in GHG mitigation scenarios included both direct and indirect sections led by unit retrofitting or construction, fuel substitution and saving, and variations in freshwater and material consumption, as calculated in equations 53-56. “X” refers to one of the M1-M5 and integrated scenarios.

$$GHGMit^X = GHGMit_D^X + GHGMit_I^X \quad (53)$$

$$WatSav^X = WatSav_D^X + WatSav_I^X \quad (54)$$

$$SO2Red^X = SO2Red_D^X + SO2Red_I^X \quad (55)$$

$$NOxRed^X = NOxRed_D^X + NOxRed_I^X \quad (56)$$

Supplementary References

1. Ministry of Housing and Urban-Rural Development of China. Code for design of thermal power plant (GB50660-2011). (2011).
2. Guo, Y., Tian, J., Chertow, M. & Chen, L. Greenhouse gas mitigation in Chinese eco-industrial parks by targeting energy infrastructure: A vintage stock model. *Environmental Science & Technology* **50,** 11403-11413 (2016).
3. U.S. Environmental Protection Agency. Catalog of CHP technologies. (2015). http://www.epa.gov/sites/production/files/2015-07/documents/catalog_of_chp_technologies.pdf.
4. PBL Netherlands Environmental Assessment Agency. Trends in global CO_2 and total greenhouse gas emissions: 2017 report. (2017). https://www.pbl.nl/en/publications/trends-in-global-co2-and-total-greenhouse-gas-emissions-2017-report.
5. National Bureau of Statistics of China. 2015 Chinese Statistical Yearbook. (2015). http://www.stats.gov.cn/tjsj/ndsj/2015/indexch.htm.
6. Ministry of Water Resources of China. 2014 Water Resource Bulletin. (2014). http://www.mwr.gov.cn/sj/tjgb/szygb/201612/t20161222_776054.html.
7. National Development and Reform Committee of China. Action plan for energy saving, emission reduction, upgrading and alteration of coal-fired electricity generation (2014-2020). (2014). http://www.zhb.gov.cn/gkml/hbb/gwy/201409/W020140925407622627853.pdf.
8. Ministry of Environmental Protection of China. Notice on the implementation of ultra-low-emission and energy-saving retrofitting of coal-fired power plants. (2015). http://www.mep.gov.cn/gkml/hbb/bwj/201512/t20151215_319170.htm.
9. National Energy Administration of China. Price report on electricity industry in China (2013-2014). (2015). http://zfxxgk.nea.gov.cn/auto92/201509/t20150902_1959.htm.
10. National Bureau of Statistics of China. 2016 Chinese Energy Statistical Yearbook. (China Statistics Press: Beijing, 2016).
11. National Bureau of Statistics of China. 2011 Chinese Energy Statistical Yearbook. (China Statistics Press: Beijing, 2011).
12. Zheng, L. *et al.* Preferential policies promote municipal solid waste (MSW) to energy in China: Current status and prospects. *Renewable & Sustainable Energy Reviews* **36,** 135-148 (2014).
13. National Energy Administration of China. The 13th five-year plan of bioenergy development. (2017). http://ghs.ndrc.gov.cn/ghwb/gjjgh/201708/t20170809_857319.html.
14. Guo, Y., Glad, T., Zhong, Z., He, R., Tian, J. & Chen, L. Environmental life-cycle assessment of municipal solid waste incineration stocks in Chinese industrial parks. *Resources, Conservation & Recycling* **139,** 387-395 (2018).

15. Ministry of Construction of China. Technical Code for Municipal Waste Incineration Projects (CJJ90-2009). (2009).

16. Guo, Y., Tian, J., Chen, L. & Chertow, M. Exploring greenhouse gas-mitigation strategies in Chinese eco-industrial parks by targeting energy infrastructure stocks. *Journal of Industrial Ecology* **22(1)** 106-120 (2018).

17. China Electricity Council. Statistical Data of Electricity Industry Development (2015). (China Electricity Council: Beijing, 2016).

18. State Council of China. Notice on advancing shutting down small-capacity thermal power units. (2007). http://www.gov.cn/zwgk/2007-01/26/content_509911.htm.

19. Jiao, S. Cost Accounting methodology of electricity generation in power plants [in Chinese]. *Gas Turbine Technology* **13(3),** 7-10 (2000).

20. China Electricity Council. Statistical Data of Electricity Industry Development (2014). (China Electricity Council: Beijing, 2015).

21. U.S. National Renewable Energy Laboratory. Life Cycle Assessment of Coal-fired Power Production. (1999). https://www.nrel.gov/docs/fy99osti/25119.pdf.

22. U.S. National Renewable Energy Laboratory. Life Cycle Assessment of a Natural Gas Combined Cycle Power Generation System. (2000). https://digital.library.unt.edu/ark:/67531/metadc724566/?q=life%20cycle%20assessment%20.

23. IKE Environmental Technology Co., Ltd, Sichuan University. Chinese Life Cycle Database V0.8. (2014). http://www.ike-global.com/archives/1094.html.

24. Liu, X. *et al.* Methods and models for developing Chinese life cycle database [in Chinese]. *Acta Scientiae Circumstantiae* **30(10),** 2136-2144 (2010).
