# Supplementary material for: Managing energy infrastructure to decarbonize industrial parks in China
Source: Nat Commun. 2020 Feb 20;11:981. doi: 10.1038/s41467-020-14805-z (PMC7033157; doi:10.1038/s41467-020-14805-z)
Supplement: Supplementary file 3 — Reporting Summary [file 41467_2020_14805_MOESM3_ESM.pdf]

nature research

Corresponding author(s): Jinping Tian

Last updated by author(s): Jan 28, 2020

Reporting Summary

Nature Research wishes to improve the reproducibility of the work that we publish. This form provides structure for consistency and transparency in reporting. For further information on Nature Research policies, see Authors & Referees and the Editorial Policy Checklist.

Statistics

For all statistical analyses, confirm that the following items are present in the figure legend, table legend, main text, or Methods section.

n/a	Confirmed	
☐	☒	The exact sample size (*n*) for each experimental group/condition, given as a discrete number and unit of measurement
☒	☐	A statement on whether measurements were taken from distinct samples or whether the same sample was measured repeatedly
☒	☐	The statistical test(s) used AND whether they are one- or two-sided<br>*Only common tests should be described solely by name; describe more complex techniques in the Methods section.*
☒	☐	A description of all covariates tested
☐	☒	A description of any assumptions or corrections, such as tests of normality and adjustment for multiple comparisons
☐	☒	A full description of the statistical parameters including central tendency (e.g. means) or other basic estimates (e.g. regression coefficient) AND variation (e.g. standard deviation) or associated estimates of uncertainty (e.g. confidence intervals)
☒	☐	For null hypothesis testing, the test statistic (e.g. *F*, *t*, *r*) with confidence intervals, effect sizes, degrees of freedom and *P* value noted<br>*Give P values as exact values whenever suitable.*
☒	☐	For Bayesian analysis, information on the choice of priors and Markov chain Monte Carlo settings
☒	☐	For hierarchical and complex designs, identification of the appropriate level for tests and full reporting of outcomes
☒	☐	Estimates of effect sizes (e.g. Cohen's *d*, Pearson's *r*), indicating how they were calculated

Our web collection on statistics for biologists contains articles on many of the points above.

Software and code

Policy information about availability of computer code

Data collection	No software is used in data collection.
Data analysis	The Vintage Stock Model is self-developed. All equations and functions used in our model are embedded into a excel file. The model is connected to source data. Besides, ArcGIS V10.4.1 is used for geographic data analysis and presentation.

For manuscripts utilizing custom algorithms or software that are central to the research but not yet described in published literature, software must be made available to editors/reviewers. We strongly encourage code deposition in a community repository (e.g. GitHub). See the Nature Research guidelines for submitting code & software for further information.

Data

Policy information about availability of data

All manuscripts must include a data availability statement. This statement should provide the following information, where applicable:

- Accession codes, unique identifiers, or web links for publicly available datasets
- A list of figures that have associated raw data
- A description of any restrictions on data availability

The source data underlying Figs. 1, 2, and 4, and Supplementary Figs. 1-4 are provided as a Source Data file. All data used for this analysis are available from publicly available sources cited or from the authors upon reasonable request. Publicly accessible data sources are basic information tables for thermal power-generating units at capacity levels of 100-225 MW, 300 MW and 600 MW (http://www.chinapower.com.cn/kjfwduibiao/20160422/4824.html and http://kjfw.cec.org.cn/kejifuwu/2013-04-07/99877.html), and lists of desulfurization and denitrification facilities of coal-fired power-generating units (http://www.mee.gov.cn/gkml/hbb/bgg/201407/t20140711_278584.htm).

Field-specific reporting

Please select the one below that is the best fit for your research. If you are not sure, read the appropriate sections before making your selection.

☐ Life sciences ☐ Behavioural & social sciences ☒ Ecological, evolutionary & environmental sciences

For a reference copy of the document with all sections, see nature.com/documents/nr-reporting-summary-flat.pdf

Ecological, evolutionary & environmental sciences study design

All studies must disclose on these points even when the disclosure is negative.

Study description	We conducted a two-phase study by targeting energy infrastructure to decarbonize Chinese industrial parks. First, a high-resolution geodatabase of energy infrastructure in 1,604 industrial parks was established. Second, a vintage stock model was developed to quantify the environmental benefits and economic costs of decarbonizing the energy infrastructure.
Research sample	1,604 China's industrial parks and their energy infrastructure
Sampling strategy	There is no sampling strategy.
Data collection	The data are recorded by authors. The open source data used for data collection are the Catalog of Industrial Parks in China (http://bgt.ndrc.gov.cn/zcfb/201803/t20180302_878802.html), and Lists of Desulfurization and Denitrification Facilities of Coal-fired Power-Generating units in China (http://www.mee.gov.cn/gkml/hbb/bgg/201407/t20140711_278584.htm). The Statistical Data of Chinese Electricity Industry are purchased from China Electricity Council. Other data are collected from on-site investigation, questionnaire, reports, and websites.
Timing and spatial scale	Mar, 2014 - Nov, 2018, China
Data exclusions	None
Reproducibility	The results can be generated by the vintage stock model we developed. If it is required for the review purpose, we could make it available for the reviewers.
Randomization	The data are not random sampling data, and randomization is not involved.
Blinding	Blinding methods are not involved in this study.

Did the study involve field work? ☐ Yes ☒ No

Reporting for specific materials, systems and methods

We require information from authors about some types of materials, experimental systems and methods used in many studies. Here, indicate whether each material, system or method listed is relevant to your study. If you are not sure if a list item applies to your research, read the appropriate section before selecting a response.

Materials & experimental systems

n/a	Involved in the study	
☒	☐	Antibodies
☒	☐	Eukaryotic cell lines
☒	☐	Palaeontology
☒	☐	Animals and other organisms
☒	☐	Human research participants
☒	☐	Clinical data

Methods

n/a	Involved in the study	
☒	☐	ChIP-seq
☒	☐	Flow cytometry
☒	☐	MRI-based neuroimaging
